# Emergency Medicine in Remote Regions

**DOI:** 10.7759/cureus.774

**Published:** 2016-09-09

**Authors:** Tia Renouf, Megan Pollard

**Affiliations:** 1 Emergency Medicine, Memorial University of Newfoundland

**Keywords:** rural medicine, antarctica, medicine at sea

## Abstract

Rural and remote places like Sable Island (Nova Scotia) or François (Newfoundland) pose a challenge in delivering both health care and appropriate education that today’s learners need to practice in a rural setting. This education can be difficult to deliver to students far from academic centers. This is especially true for learners and practitioners at offshore locations like ships, oil installations, or in the air when patients are transported via fixed wing aircraft or helicopter. The following editorial provides a snapshot of the setting and the challenges faced while working as a physician on a ship, in remote regions.

## Editorial

I write this editorial from the *Akademik Ioffe*, the ship that held my heart for 15 years since I was last her doctor in Antarctica. Soon she will take me along with 102 passengers to beautiful, rural but remote places like Sable Island (Nova Scotia) and François (Newfoundland). Both places are magical to visit, but in the longer term, they provide us with a challenge in terms of delivering both health care and the necessary education for today’s learners to practice in a rural setting. Unlike seasoned physicians, newer students may rely more upon sophisticated technology for diagnosis and treatment, which is rarely found in rural and remote areas. As the ship’s doctor, I hope this voyage does not present the kinds of clinical challenges that characterize practice in faraway places. If somebody gets sick aboard ship or is injured in François where there is no road, it is a far different proposition to care for them than in the academic emergency room, where I normally work.

Delivering rural and remote health professions education (HPE) is similarly challenging. The mission statement of the medical school at Memorial University of Newfoundland (MUN) states that students should receive the same quality of education no matter where in the province they are learning. In reality, this can be difficult for students who are far from an academic center. This is particularly true for learners and practitioners at offshore locations like ships and oil installations and in the air when patients are transported via fixed wing aircraft or helicopter. The challenges vary tremendously, from communicating effectively [1], through flawlessly performing seldom-used critical procedures in an emergency, to the logistics of evacuation, particularly in harsh environments where ice, sea conditions, and weather are serious considerations. It is often impractical and even potentially unsafe for learners to experience these extreme environments firsthand; hence, we use simulation as a vehicle to provide this kind of training. The simulation may be especially useful for learning how to manage low-frequency, high-stakes events that are likely to be encountered in remote places. Further, recognizing the importance of direct exposure to rural medicine, distributed medical schools like Memorial University provide training opportunities in some very rural and remote environments throughout Labrador and in Nunavut. Many universities and national societies like the SRPC (Society of Rural Physicians of Canada) also offer rural/remote training courses. SRPC's journal, the Canadian Journal of Rural Medicine (CJRM) produces brief communications on doing occasional procedures.

Finally, we are underway, and my thoughts return to the journey. The heavy anticipation and wait for departure lift as I feel the bow thrusters pushing us away from the Halifax pier. The ship’s crew is relieved to be moving too, for being in motion defines the voyage.

**Figure 1:**
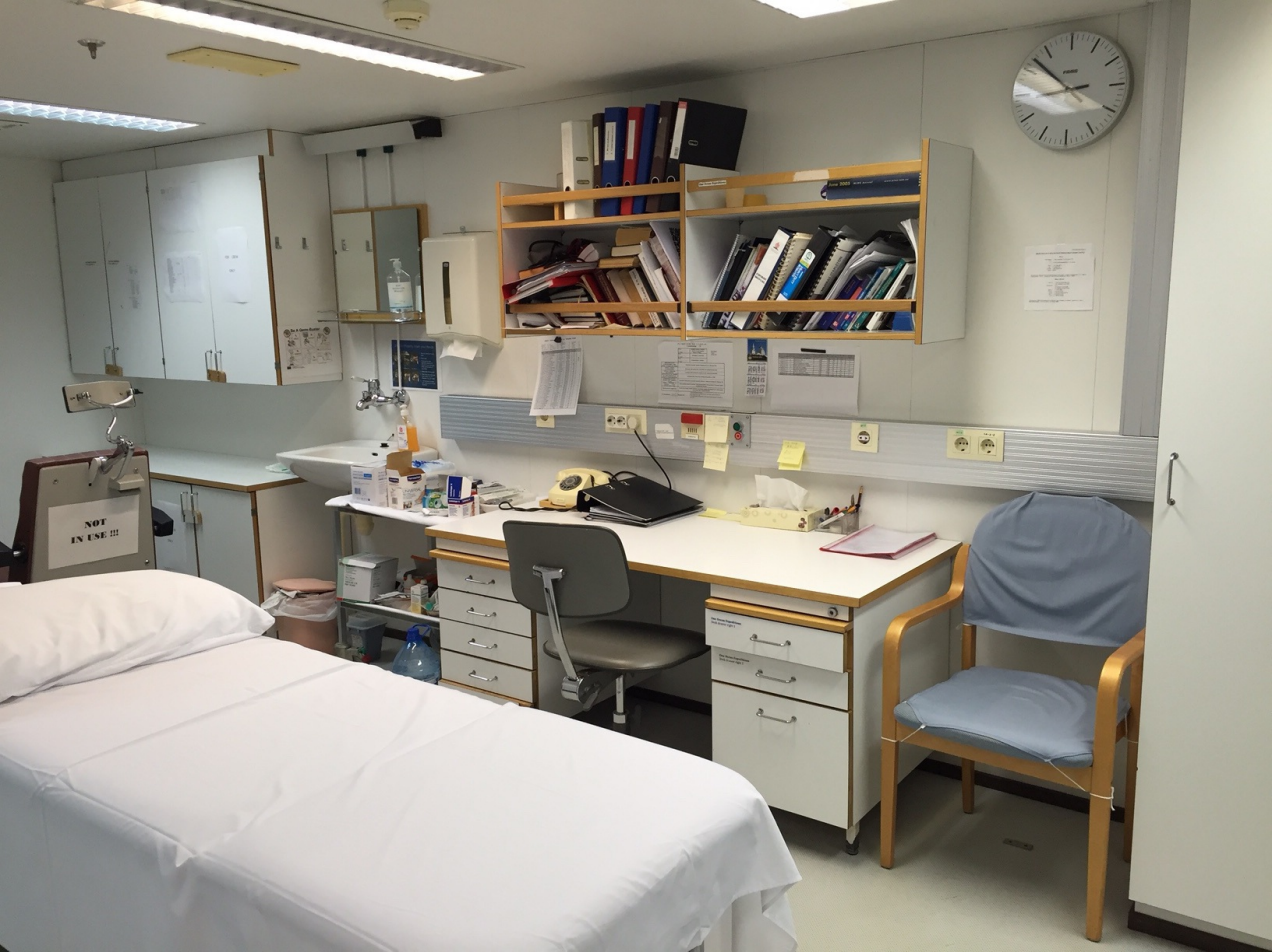
The ship's infirmary

**Figure 2:**
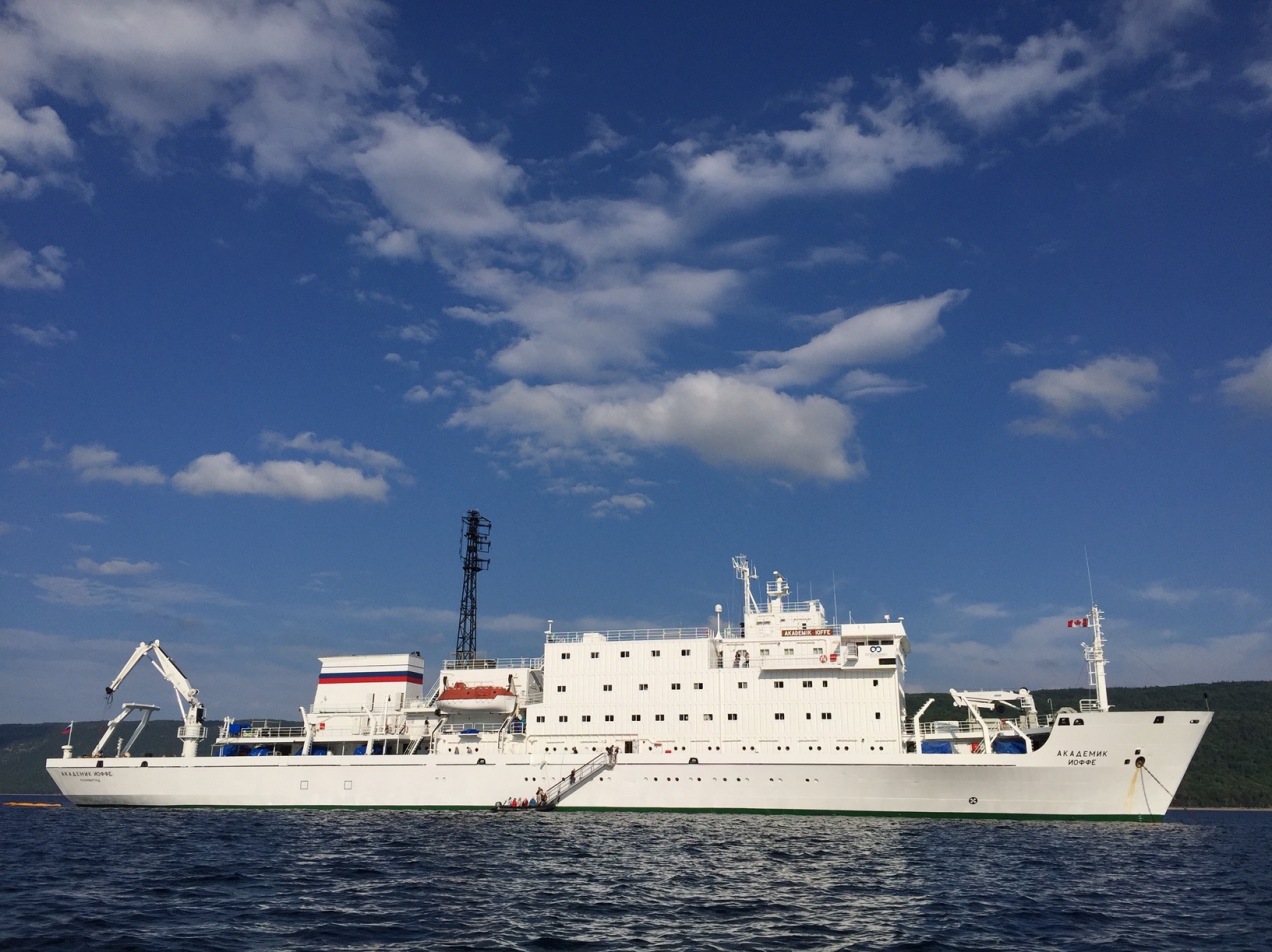
Zodiacs are lowered via the crane at the stern

I have already checked the infirmary’s supplies (see Figure 1), then put tape over their cabinets, lest a turbulent sea scatters and breaks glass ampules of drugs. “Keep one hand on the ship” is my mantra to the passengers, many of whom are elderly and at risk of falling. When the ship is at anchor, they will descend the gangway several times daily into Zodiacs that will bring them ashore. Once they are on land they will often hike or bicycle. Some will kayak. Fortunately, injuries are few. The crew is an expert at making sure these operations run smoothly. I once watched them seamlessly transfer 12 passengers from a rapidly deflating Zodiac to another boat, in frigid Antarctic waters (Figure 2). A carnivorous leopard seal, (known as the polar bear of the Antarctica because it has no predators), had delivered a toothy *coup de grâce* to the Zodiac’s pontoon. For more mundane emergencies, I carry a variety of supplies and a defibrillator and remain in constant radio contact with the crew and the bridge.

The ships’ logs reveal a variety of illnesses and injuries at sea [2]. So far on this voyage, I have had some suturing to do and evacuated one passenger with a potentially serious condition. On a previous voyage in a polar region, I treated a severe exacerbation of emphysema, which normally calls for non-invasive ventilation and intensive care consultation. On the ship, all I had were puffers, oral antibiotics, and prednisone.

Like the ship’s doctors, physicians working in rural or remote locations are occasionally required to treat critical illnesses using the few tools they have at hand. A trauma patient, for example, may need urgent intubation. A rural physician may only have a basic intubation equipment and may not have performed an intubation or managed a serious trauma for many months or years. However, a rural physician without a replacement finds it difficult to leave their community to take refresher courses. The question arises that unless the course was very recent, would the rural physician have retained the necessary skills and confidence to manage a critically injured patient? Many centers can deploy helicopters carrying experts to retrieve critically ill patients, but the helicopter operation is weather-dependent. These are the realities of rural and remote medical care in areas of the world with harsh climates, such as polar regions, where unfavorable weather and ice conditions can forestall patient transportation to definitive care indefinitely.

Learners must experience these realities and learn to work with the resources available to them. In some instances, they may have access to helpful resources. In locations with an appropriate telecom infrastructure, for example, telemedicine allows an expert to guide procedures from afar. Also, simulation may be used to deliver high-stakes, low-frequency teaching, both to learners and graduate physicians who must maintain their skills. We typically associate simulation with computerized human mannequins, but mannequins can be impractical to transport and support in rural/remote locations. Using locally available materials may be a particularly advantageous alternative. For example, while teaching emergency skills at one of MUN’s rural/remote locations, we built a chest tube task trainer with hard foam found at a construction site, an empty bankers box, and pork ribs from a local grocer. In Ethiopia, participants in one study used shoelaces to practice surgical throws [3]. Shoelaces make ideal suture material; ubiquitous in local markets, they do not dry out and/or break like commercially available material, whose alcohol preservative evaporates over time. Colleagues at the Justinian University Hospital in Cap-Haitien, Haiti, devised a laparoscopic surgery task trainer from a variety of inexpensive local materials [4]. If sound teaching practices are employed, the learning is just as good using these simple tools [5] as with the computerized human mannequins described above. Tools made with local materials can be readily assembled, need no special transport, and can be left at rural/remote teaching sites for continued practice.

**Figure 3:**
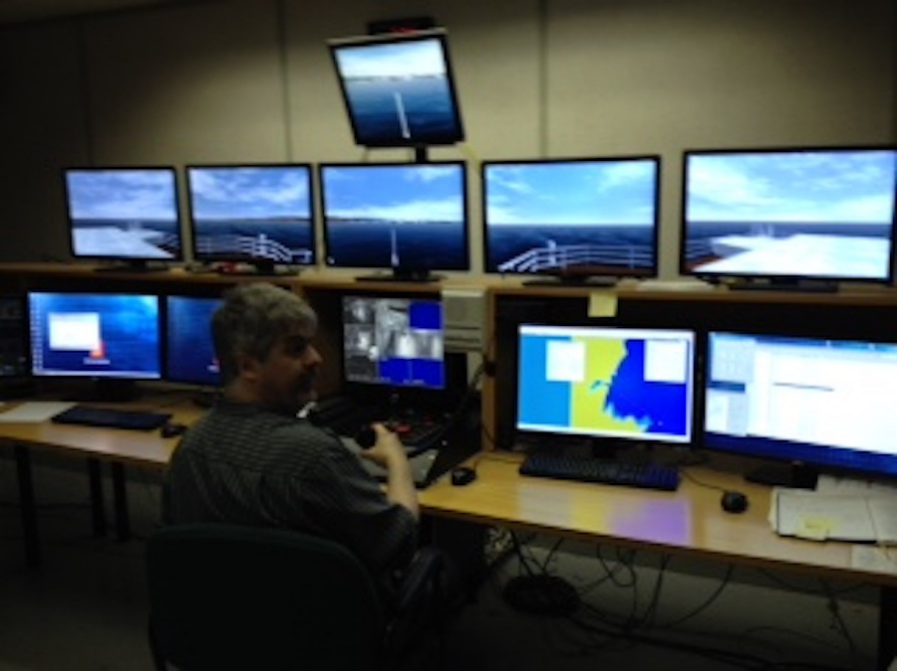
View of the ship simulator at the Marine Institute in St. John's, NL

A simulation-augmented HPE means writing scenarios, which could be a time-consuming task, particularly for rural/remote physicians who may work alone or with a single colleague. On a previous voyage when the ship had a long sea day and the crew had some time, we practiced how to manage a simulated kayak accident. On my return, we will reproduce the accident in our university’s marine simulator (Figure 3) to manipulate the sea state, wind conditions, and visibility. The work is intended for publication in an open access journal, making it a freely accessible teaching tool for clinician educators.

The *Ioffe* has finished her journey and is at anchor in Louisbourg (Nova Scotia). The crew is replenishing ship supplies while her navigator is charting a course for Iqaluit, the starting point for the next voyage: through the North West Passage and eventually to Greenland. Another ship’s doctor will replace me and keep watch over another 100 or so passengers. They will sail to iconically beautiful and remote places and perhaps encounter medical emergencies. May fair winds prevail!
